# Bleeding Versus Thrombotic Tendency in Young Children With Beta-Thalassemia Major

**DOI:** 10.7759/cureus.20192

**Published:** 2021-12-06

**Authors:** Shalini Singh, Geeta Yadav, Rashmi Kushwaha, Mili Jain, Wahid Ali, Nishant Verma, Shailendra P Verma, U. S. Singh

**Affiliations:** 1 Department of Pathology, King Georges Medical University, Lucknow, IND; 2 Department of Paediatrics, King Georges Medical University, Lucknow, IND; 3 Department of Clinical Oncology (Clinical Hematology), King Georges Medical University, Lucknow, IND

**Keywords:** beta-thalassemia major, hepatic dysfunction, activated partial thromboplastin time, prothrombin time, hyperferritinemia, protein s, protein c, platelet aggregation, bleeding, thrombosis

## Abstract

Introduction

Bleeding and thrombotic events are known to occur in beta-thalassemia major (BTM) patients and have been attributed to hepatic iron overload associated with multiple blood transfusions. We evaluated hemostatic parameters in children with BTM who had no previous history of bleeding or thrombotic episodes.

Materials and Methods

Hemostatic parameters including prothrombin time (PT), activated partial thromboplastin time (APTT), platelet aggregation, protein C and S, iron profile, and liver function tests were evaluated in 54 children (median age = 12 months, age range = 4-144 months) with BTM and 15 age and sex-matched controls.

Results

The mean PT and APTT of patients were significantly higher (*P*=0.016 and *P *<.001) than that of controls. Mean protein C, protein S activity and platelet aggregability with adenosine 5-diphosphate (ADP) as an agonist in patients were significantly lower (*P *<.001, *P *<.001 and *P*=0.007, respectively) than that in controls. Mean serum ferritin in BTM children was not significantly elevated to be associated with hepatic dysfunction.

Conclusion

Deranged hemostatic parameters indicative of bleeding and thrombotic tendencies are observed in BTM children from an early age and may not be solely due to hyperferritinemia-associated hepatic dysfunction. Despite the presence of deranged hemostatic parameters, a state of balance exists between bleeding and thrombosis, and an imbalance may lead to bleeding or thrombotic events at a later age.

## Introduction

Beta-thalassemia is the commonest hereditary hemoglobinopathy in India with a prevalence of approximately 3%-4% [1]. Nearly 8,000-12,000 children are born each year with thalassemia major which is associated with significant morbidity and mortality and poses a considerable health problem [2]. With standard treatment protocols which include multiple blood transfusions, erythroid maturation agents, iron chelation, splenectomy, and allogenic bone marrow transplantation, there has been an improvement in the average life span; however, multiple blood transfusions are associated with iron deposition in the liver, heart and endocrine organs, which is the cause of 90% mortality in thalassemia patients. Recent studies have also observed profound hemostatic changes in thalassemia and have attributed these changes to iron overload owing to multiple blood transfusions [3]. Hemostatic abnormalities described include bleeding, epistaxis, subarachnoid hemorrhage, deep vein thrombosis, pulmonary thromboembolism, and stroke [4,5]. Little is known about the time of onset, triggering factors, and pathophysiology of bleeding and thrombotic events in patients with thalassemia. In this study, we aimed to determine subclinical deranged hemorrhagic or thrombotic parameters in very young children (median age 12 months, age range = 4-144 months) with beta-thalassemia major (BTM) with a lesser number of blood transfusions.

## Materials and methods

This was an analytical cross-sectional study done over a period of one year. The study was approved by the Institutional Ethics Committee (207/Ethics/R.Cell.18; reference code 89th ECM II B Thesis/P21). Written informed consent was obtained from the parents of all patients included in the study. A total of 54 pediatric patients with a confirmed diagnosis of BTM on HPLC studies (done on Bio-Rad, VARIANT II hemoglobin testing system) and/or hemoglobin subunit beta (HBB) gene mutation studies were enrolled in the study. Fifteen age and sex-matched healthy children attending the vaccination clinic were recruited as a control group. A detailed history was obtained, and a clinical examination was done on all patients. Patients with hepatitis, overt liver failure, cardiomyopathy, family history of bleeding or thrombotic disorders, or on aspirin therapy were excluded.

Blood samples were collected from an antecubital vein under aseptic conditions before blood transfusion (which on average took place every 25-30 days) and dispensed in plain (2 mL), K3EDTA (2 mL), and 3.2% sodium citrate (6 mL) vials. Blood in a plain vial was centrifuged at 2,500 rpm for the separation of serum. Serum was stored at -20 ^0^C. Iron overload was assessed by estimating serum ferritin, serum iron, and serum total iron-binding capacity (TIBC). Serum ferritin was assessed using Invitrogen™ Ferritin Human ELISA Kit (Thermo Fisher Scientific, Waltham, MA, USA). Serum iron and serum TIBC were estimated using, Iron & TIBC kit (Tulip Diagnostics, Bambolim, Goa, India). The severity of liver damage was assessed by serum bilirubin, serum aspartate transferase (AST), and serum alkaline phosphatase (ALP). Serum bilirubin (total and direct), Serum AST, and serum ALP were estimated on a semi-automated chemical analyzer (Selectra Pro-XL, ELITechGroup Clinical Systems, Netherlands) using Q-line^TM^ BILIRUBIN (TOTAL & DIRECT), Q-line® S^+^ AST/GOT 4+1 SL, and Q-line^TM^ S^+^ ALT/GPT 4+1 SL kits (Q-Line Biotech Private Limited, New Delhi, India), respectively. Complete blood count was done on K3EDTA blood by hematology autoanalyzer (ADVIA 2120 system, Siemens Healthcare Diagnostics, Tarrytown, NY, USA). Blood smears were also made and stained with Leishman stain for morphological evaluation. Coagulation and thrombotic assays including prothrombin time (PT), activated partial thromboplastin time (APTT), protein C and protein S activity assay were performed on blood collected in 3.2% sodium citrate vials on fully automated STA Satellite®Hemostasis System (Diagnostica Stago Inc., France) using STA®-Neoplastine® CI 5 kit, STA®-PTT Automate 5 kit, STA®-Staclot Protein C kit, and STA®-Staclot Protein S kit, respectively. Platelet aggregation studies were done in platelet-rich plasma (PRP) using adenosine 5-diphosphate (ADP) as an agonist on Chrono-Log® Optical Aggregometer (Chrono-Log Corporation, Havertown, PA, USA). PRP) was obtained by centrifugation of blood collected in 3.2% sodium citrate vials at room temperature (20 ^0^C- 25 ^0^C) for 5 min at 1,000 rpm. PRP was removed carefully. Platelet Poor Plasma (PPP) was obtained by centrifuging the remaining blood (after separation of PRP) for 10 min at 2,000 rpm and 5 μM/mL working solution of ADP previously stocked at -80 ^0^C was used as an agonist. The aggregometer was switched on about 30 min before the tests to be performed to allow the heating block to warm up to 37 ^0^C. 500 μL of PRP was pipetted into a cuvette which was placed into the heating block for incubation. PRP was warmed up to 37 ^0^C for 2 min and then 5 μL ADP was added. Change in absorbance was noted at 5 min or until the response reached a plateau (whichever was sooner). Precautions were taken to complete platelet aggregation studies within four hours of sample collection. The results were expressed as the maximal impedance change in percentage (%).

Statistical analysis

Statistical Package for Social Sciences (SPSS) software version 23.0 (SPSS Inc., Chicago, IL, USA) was used for statistical analysis of data. The values were represented in percentage (%) and Mean ± Standard deviation (SD). The student’s t-test was used to compare parameters in different subgroups. The Chi-square test was used for categorical variables. The difference was considered significant if the p-value was <.05.

## Results

Fifty-four children with a confirmed diagnosis of BTM based on HPLC studies and/or HBB gene mutation studies were included in this study. Of 54 BTM children included in this study, there were 44 males and 10 females (Male: female ratio = 4.4:1). The median age of all children was 12 months (age range = 4-144 months). The diagnosis of BTM has obtained splenomegaly (spleen palpable below the left costal margin) was observed in 49/54 (90.74%) children with BTM. The mean spleen size below the costal margin was 3.79 ± 3.06 cm and none of the patients included in this study were splenectomized. Blood transfusions were required in all patients. The mean number of blood transfusions per patient was 5.26 ± 4.86 and only 12 (22.22%) children had received more than five blood transfusions as a majority were recently diagnosed cases. Mean nucleated RBC’s/100 WBC’s and mean platelet count were 15.70 ± 18.26 and 220 ± 86 x10^9^/L, respectively.

Mild derangements in liver function parameters including a mild increase in mean total serum bilirubin (1.63 ± 0.60 mg/dL), unconjugated bilirubin (0.86 ± 0.45 mg/dL), AST (45.23 ± 32.72 IU/L), and ALP (441.67 ± 190.84 IU/L) were observed among children with BTM in this study. Mild derangements in serum iron profile including a mild increase in mean serum ferritin (815.38 ± 789.73 ng/mL) and mean serum iron (140.62 ± 42.57 µg/dL) and mild decrease in mean serum TIBC (229.20 ± 32.64 µg/dL) was observed among children with BTM in this study. The clinical, biochemical, and hematological parameters of the study population (n= 54) are tabulated in Table 1.

**Table 1:** Clinical, biochemical, and hematological parameters of the study population (n = 54) NRBC - nucleated RBC; AST - aspartate transferase; ALP - alkaline phosphatase; TIBC - total iron-binding capacity

Parameter (units)	Values in study patients (Mean ± SD)	Normal values [6]
Clinical parameters		
Spleen size below costal margin (cm)	3.79 ± 3.06	-
Hematological parameters		
NRBC (per 100 WBC)	15.70 ± 18.26	-
Platelet count (x10^9^/L)	220 ± 86	150-400
Biochemical parameters		
Total Serum bilirubin (mg/dL)	1.63 ± 0.60	0.1-1.2
Indirect serum bilirubin (mg/dL)	0.86 ± 0.45	0.1-1.0
Serum AST (IU/L)	45.23 ± 32.72	8-33
Serum ALP (IU/L)	441.67 ± 190.84	20-130
Serum ferritin (ng/mL)	815.38 ± 789.73	7-140
Serum iron (μg/dL)	140.62 ± 42.57	60-150
TIBC (μg/dL)	229.20 ± 32.64	250-400

The mean PT of patients (14.6 ± 1.24 seconds) was significantly higher (*P *=.016) than that of controls (13.93 ± 0.59 seconds). PT was prolonged (>17 seconds) in 5.55% (3/54) of all patients. The mean APTT in patients (36.41 ± 7.42 seconds) was significantly higher (*P *<.001) than the mean APTT in controls (27.33 ± 2.94 seconds). APTT was prolonged (>37 seconds) in 37.03% (20/54) of all BTM patients. The difference in APTT between patients and controls was more pronounced as compared to PT. The mean protein C activity in patients (64.81 ± 17%) was significantly lower (*P* <.001) than the mean protein C activity in controls (102.67 ± 19.21%). Protein C activity was decreased (<65%) in 46.29% (25/54) of BTM patients in this study. The mean protein S activity in patients (62.3 ± 20.91%) was significantly lower (*P* <.001) than the mean protein S activity in controls (106.87 ± 16.56%). Protein S activity was decreased (<65%) in 57.40% (31/54) BTM patients in this study. The mean platelet aggregability on optical aggregometry using ADP as an agonist was significantly lower (*P* =.007) in patients (42 ± 24.28%) when compared to controls (59.00 ± 12.00%). 29.6% (16/54) BTM patients in this study showed platelet hypoaggregation. Table 2 compares hemostatic parameters between β-thalassemia major patients and controls.

**Table 2:** Comparison of hemostatic parameters (mean ± SD) between β-thalassemia major patients and controls PT - prothrombin time; APTT - activated partial thromboplastin time

	β--thalassemia major (n= 54)	Controls (n= 15)	Significance of difference	
	Mean ± SD	Mean ± SD	t	*P*’
Bleeding parameters				
PT (seconds)	14.6 ± 1.24	13.93 ± 0.59	2.15979	.016971
APTT (seconds)	36.41 ± 7.42	27.33 ± 2.94	4.86441	* *< .001
Thrombotic parameters				
Protein C activity (%)	64.81 ± 17.0	102.67 ± 19.21	-6.26838	< .001
Protein S activity (%)	62.3 ± 20.91	106.87 ± 16.56	-6.57289	< .001
Platelet parameters				
Platelet aggregation (%)	42 ± 24.28	59.00 ± 12.00	-2.48057	.007664

## Discussion

The presence of significant hemostatic changes (either bleeding or thrombosis) has been documented in children with BTM [4,5,7]. These changes have been attributed to hepatic dysfunction caused by an iron overload due to repeated blood transfusions [4,7,8]. Pronounced hepatic dysfunction due to hepatic iron overload usually does not occur in BTM patients until serum ferritin levels cross 2,000 ng/mL [9]. In this study we evaluated laboratory hemostatic parameters in relatively younger children with BTM who had no history of bleeding or thromboembolic episodes and had received lesser number of blood transfusions.

This study included 54 BTM patients of relatively younger age (median age = 12 months; age range = 4-144 months). Mild elevation of mean serum ferritin and mean serum iron levels which were 793.27 ± 696.18 ng/mL and 139.41 ± 43 μg/dL, respectively, were observed in children with BTM in this study. The mean serum ferritin level was relatively lower in our study as compared to previous studies. Naithani et al. observed bleeding manifestations in 29.6% of their patients with BTM (n=54, mean age = 9.68 ± 4.3 years). The mean serum ferritin and iron levels observed in their study were 3,709 ± 1,625 ng/mL and 190 ± 89 μg/dL, respectively [4]. Borgna-Pignati et al. observed thromboembolic events in 3.95% of their BTM patients (n=685, mean age=16 years, age range = 6-28 years). The mean serum ferritin levels observed in their study was 5,405 ng/mL (range = 2,160-11,000 ng/mL) [10]. In our study, 35.18% (19/54) patients had serum ferritin level >1000 ng/mL and only 5.5% (3/54) patients had serum ferritin level >2,000 ng/mL. This can be explained by the relatively lesser requirement of mean number of blood transfusions (5.26 ± 4.86) due to the relatively younger study population in this study.

Thrombocytopenia (platelet count < 150 x 10^9^/L) was seen in 13.2% of patients in our study. The mean platelet count of patients in our study was 220 ± 86 x 10^9^/L (range = 110-492 x 10^9^/L). Thrombocytopenia has been observed in 10%-40% of BTM patients in different studies [4,11,12]. Thrombocytopenia in β-thalassemia patients has been attributed to hypersplenism, hepatic dysfunction because of iron overload and oral iron chelator (deferipone) therapy for iron overload [4,13]. A chronic consumptive state due to chronic activation of the intrinsic coagulation cascade owing to multiple transfusions has also been proposed to be responsible for thrombocytopenia in BTM [12].

PT and APTT were prolonged in 5.55% and 37.03% of our patients with BTM. Prolongation of PT and APTT in BTM patients has been attributed to hepatic parenchymal damage by iron overload [14], chronic activation of the intrinsic coagulation and/or kallikrein systems following intravascular hemolysis and multiple blood transfusions [12].

Platelet hypoaggregation was observed in 29.6% of non-splenectomized BTM children in our study on optical aggregometry against ADP as agonist. None of the patients in our study had markedly reduced platelet counts so as to be a limiting factor for platelet aggregation studies. Platelet hypoaggregation has been observed in 44%-66% of non-splenectomized BTM patients in other studies [15-17]. Various reasons have been proposed for platelet hypoaggregation in non-splenectomized BTM patients. Chronic in vivo activation of platelets due to release of ADP from hemolyzed RBCs renders platelets refractory to further stimuli in vitro [18]. Tissue hypoxia due to chronic anemia may damage the endothelium causing interaction of platelets with the vessel wall. This results in the formation of circulating aggregates by the more active platelets while the less active ones are detected in vitro as poorly aggregable [16,19]. Orudzhev et al. observed increased levels of circulating antiplatelet antibodies, immunoglobulins, and variable-sized immune complexes in their β-thalassemia patients and suggested their role in causing increased platelet disaggregation with resultant in vitro hypoaggregation of platelets [20]. On the other hand, hyperaggregation along with incidences of thrombosis has been observed in splenectomized BTM or heterozygous thalassemia patients [19,21]. None of the patients in our study were splenectomized and we did not observe platelet hyperaggregation or thrombotic episodes in any of the patients in our study. Increased levels of free alpha globin chains in splenectomized thalassemia patients have been proposed to cause oxidative damage to integral and cytoskeletal proteins of RBCs thereby exposing phosphatidyl ethanolamine and phosphatidylserine on the surface of RBCs. The exposed phosphatidylserine leads to the conversion of prothrombin to thrombin and thereby causes platelet activation [7,22].

The mean protein C activity (64.81 ± 17%) and the mean protein S activity (62.3 ± 20.91%) in β-thalassemia patients in our study was significantly lower than the mean protein C activity (102.67 ± 19.21%) and the mean protein S activity (106.87 ± 16.56%) of controls in our study. Hepatic dysfunction owing to iron overload, a chronic hypercoagulable state and abnormal thalassemic RBCs have been proposed as the cause of reduced protein C and S levels in BTM patients. Capellini et al. suggested that protein C and S are vitamin K-dependent plasma proteins synthesized in the liver and hepatic dysfunction owing to iron overload may be the cause for their reduced levels in β-thalassemia patients [8]. Hassan et al. proposed that protein C has affinity for phosphatidylserine and other negatively charged phospholipids, which are abnormally present on outer membrane of thalassemic RBCs, and this may be responsible for the decreased levels of protein C in β-thalassemia patients [23]. Absodera et al. suggested that the most probable cause for decreased protein C and protein S levels in β-thalassemia patients seems to be the increased consumption of these anticoagulants in their attempt to control the chronic activation of the coagulation system [24].

Our study has certain limitations. The study was a cross-sectional study and follow-up of patients was not done to assess for the development of bleeding or thrombotic episodes in children with BTM. The effect of splenectomy on hemostatic parameters could not be assessed as none of BTM children in our study had undergone splenectomy. A global assay of all hemostatic parameters including D-dimer, von Willebrand factor (vWF) antigen, factor VIII levels, lupus anticoagulant, antithrombin III, etc. and ultrasonography to assess for thrombosis was not done in our study.

## Conclusions

Deranged hemostatic parameters can exist in otherwise healthy children with BTM right from infancy and this may not be solely contributed by hepatic dysfunction or multiple blood transfusions. In children with BTM, a state of balance exists between bleeding and thrombosis in vivo despite the presence of deranged hemostatic parameters and an imbalance may lead to either bleeding or thrombosis at a later age. Further studies are required to predict the probability and the triggering events for thrombosis or bleeding manifestations in children with β-thalassemia major.
